# Sensory characteristics and consumer liking of sausages with 10% fat and added rye or wheat bran

**DOI:** 10.1002/fsn3.126

**Published:** 2014-06-02

**Authors:** Louise Margrethe Arildsen Jakobsen, Stine Vuholm, Margit Dall Aaslyng, Mette Kristensen, Karina Vejrum Sørensen, Anne Raben, Ursula Kehlet

**Affiliations:** 1 Department of Nutrition, Exercise and Sports, Faculty of Science, University of CopenhagenRolighedsvej 30, Frederiksberg C, DK-1958, Denmark; 2 Danish Meat Research Institute, Danish Technological InstituteMaglegaardsvej 2, Roskilde, DK-4000, Denmark

**Keywords:** Bran, consumer liking, dietary fiber, sausage, sensory characteristics

## Abstract

Improving the nutritional profile of sausages through the addition of dietary fiber might affect appetite, sensory characteristics, and liking differently depending on the fiber source. This study investigates the sensory characteristics and consumer acceptance of sausages with 10% (w/w) fat and added rye or wheat bran. Sensory descriptive attributes (odor, appearance, texture, and flavor) of rye bran sausage (RBS) and wheat bran sausage (WBS) were evaluated by a trained sensory panel (*n* = 9). A sausage with wheat flour (WFS) and two commercial 20% (20%S) and 10% (10%S) (w/w) fat sausages were also included. Liking was investigated in consumer tests with two Danish target groups (49 children aged between six and nine and 24 parents). RBS and WBS were similar with regard to their sensory descriptive attributes, but the structure of these sausages was coarser and the color was more brown than the other sausages. RBS was similar to the commercial 10%S with regard to several sensory attributes and liking, whereas WBS was the least juicy, had a higher intensity of cereal odor and flavor, and the lowest liking.

## Practical Applications

Information obtained in the present study can be instrumental in enhancing the development of successful, healthy alternatives to potentially unhealthy meat products and therefore has practical use in industrial meat product development. The study provides knowledge on how the addition of dietary fiber from rye or wheat bran to sausages affects sensory properties and consumer liking. This unique combination of objectively evaluated sensory properties and subjective consumer liking shows that the addition of rye bran represents a promising approach towards improving the health benefits of sausages while maintaining consumer liking. The results open up the possibility of adding viscous dietary fiber to sausages, thereby obtaining a healthy product that is accepted by the consumers. Viscous fibers are unique in that they appear to have a more profound effect on the satiety response. In this respect, it might also be relevant to investigate other sources of viscous dietary fiber besides rye bran.

## Introduction

Increasing focus on health and nutrition has led to growing consumer demand for healthier food products. Meat and meat products are particularly interesting with regard to appetite and weight management, since they contain a high proportion of protein. High-protein diets have been shown to enhance satiety when compared with low-protein diets (Vandewater and Vickers 1996; Halton and Hu 2004; Weigle et al. 2005; Paddon-Jones et al. 2008; Gilbert et al. 2011; Te Morenga and Mann 2012). Sausages represent a well-known food product that is popular amongst many consumers because of their high palatability. However, nutritionally, sausages do not normally contribute to a healthy diet, since they contain up to 20–25 g fat per 100 g, resulting in a very high energy density. The energy content is often associated with the taste of the product, and the fat content contributes to a specific mouth-feel (Drewnowski 1998; Szczesniak 2002). Consumers are not willing to compromise when it comes to texture, taste, and flavor, and this poses a challenge for the food industry in terms of developing new healthy food products, since consumers will only repurchase a product if it tastes good (Chambers et al. 1996; Drewnowski 1998). In this respect, soluble dietary fiber might have functional properties in food products, such as increased water-holding capacity and gelation, which affect the texture and contribute to maintaining texture of fat-reduced products (Elleuch et al. 2011). Incorporating functional ingredients such as dietary fiber into meat products is also a way of providing desirable nutritional properties. Dietary fiber has different effects on appetite, energy intake, and body weight depending on solubility and viscosity (Wanders et al. 2011). Dietary fiber that forms viscous solutions has been shown to decrease appetite sensation and energy intake in short-term trials (Wanders et al. 2011).

Several studies have investigated the effects on sensory properties of adding oat bran (Chang and Carpenter 1997), purified oat or wheat fiber (Cofrades et al. 2000; Szczepaniak et al. 2005) or oat, wheat, or rye bran (Yılmaz and Dağlıoğlu 2003; Yılmaz 2004, 2005) to sausages or other meat products such as meatballs. These studies show that adding the crude bran fraction generally contributes to a greater change in sensory properties than adding purified fiber in the form of fiber-rich powders. However, the fat and water contents of meat products are also very important factors with regard to texture. A direct comparison of the effects of adding rye or wheat bran to sausages has not yet been conducted. These two sources of dietary fiber are interesting, because they contain different proportions of soluble and nonsoluble dietary fiber. Arabinoxylans are the most dominant type of fiber in both rye and wheat bran, though the proportion of soluble arabinoxylans is highest in rye bran (Nyström et al. 2008; Belitz et al. 2009).

Vuholm et al. (2014) documented that meals containing sausages with wheat or rye bran increased the feeling of fullness and satiety and decreased hunger and the sensation of prospective energy or food consumption when compared with a meal containing sausages without dietary fiber. There was a tendency toward a greater satiating effect of the meal with sausages containing rye bran compared with sausages with wheat bran. Interestingly, the different meals did not affect palatability ratings, except for odor, which was rated as slightly more appetizing for the meal with rye bran sausages (RBS) compared with the meal with wheat bran sausages (WBS). The study by Vuholm et al. (2014) was made in parallel to the present study and palatability was not the main focus, as the sausages were part of a meal and not evaluated individually. These results show that the addition of dietary fiber has the potential to improve the nutritional profile of sausages, and it is likely that this can be done without having an adverse effect on the palatability. However, it is unclear how dietary fiber contributes to changes in texture, appearance, taste, odor, and flavor, as well as consumer liking.

It is very important that the consumers like the healthy alternative product, since otherwise they will not eat it and will therefore not receive the nutritional benefits of the dietary fiber. The aim of this study was to investigate whether the addition of dietary fiber via wheat or rye bran to sausages with 10% w/w fat could affect sensory properties and consumer liking.

## Materials and Methods

### Sausages

The sausages included in this sensory study were: WBS, RBS, wheat flour sausage (WFS), and also two commercial sausages from the producer Stryhns A/S: a fat-reduced 10% w/w fat sausage containing potato fiber (10%S) and a standard 20% w/w fat sausage (20%S). The two commercial sausages were included in the study because they represent the competing products on the Danish market.

The WBS and the RBS were also used in the appetite study described by Vuholm et al. (2014), and a WFS was used as a control sausage without dietary fiber in order to investigate the satiating effects of bran addition. The WFS was therefore also included in this study.

The sausages were produced by the Danish Meat Research Institute (DMRI) and were based on a standard recipe for frankfurters with 60% boneless pork shoulder (ESS Food no. 1313), 5% fat, and 1.5% salt. Instead of water, 4.55 and 6.2% rye (Valsemøllen, Esbjerg, Denmark) or wheat bran (Valsemøllen) were added to the recipes for RBS and WBS, respectively. The aim was to include the same percentage of dietary fiber in the two sausages based on information from the company providing the bran. In the recipe for WFS, 4.2% of refined wheat flour (Guldvang, Aldi, Germany) was added instead of water. After drying, the sausages were packed in modified atmosphere (30% CO_2_/70% N_2_) and stored at 5°C for up to 6 weeks prior to use. The five sausages and their nutritional composition are described in Table 1.

**Table 1:** Nutrient content of the test sausages based on nutritional calculations using data from recipes (WBS, RBS, WFS), and declarations (20%S, 10%S) respectively (WINFOOD® 4.9).

	WBS	RBS	WFS	20%S	10%S
Fiber source	Wheat bran	Rye bran	–	–	Potato
Energy (kJ)	771	723	714	1050	620
Protein (g)	13.9	13.4	13.4	12	10
Carbohydrate (g)	5.6	4.3	4.5	5	8
Dietary fiber (g)	3.2	2.4	0.4	–	2
Fat (g)	11.2	10.9	11.0	21	9

Values are given per 100 g. RBS, rye bran sausage; WBS, wheat bran sausage; WFS, wheat flour sausage; 10%S, 10% fat sausage; 20%S, 20% fat sausage.

### Sensory profiling

The sensory profiling was performed at the sensory laboratory at DMRI in Roskilde, Denmark, which is accredited to the standard DS/EN ISO/IEC 17025:2005. The method used was inspired by the Quantitative Descriptive Analysis described by Meilgaard et al. (2007). The sensory panel consisted of nine trained female panelists aged between 42 and 70 and with 2–32 years of experience in the sensory assessment of meat and meat products.

Panel training was performed in two sessions, each lasting 3–4 h, over a period of 2 days prior to the sensory profiling and took the form of a combination of group discussion and individual rating in the sensory laboratory. To avoid bias, a passive panel leader, who was aware of the aim of the sensory profiling, conducted the training. Since the panelists had already been trained in the evaluation of meat products, the training primarily focused on the description of the sausages, the establishment of a relevant set of attributes, standardization of the handling of the sausages, and finally the use of the scale. No reference samples were considered necessary for the profiling. The test sausages were included during the training, although the panelists were not informed of the content of the sausages and they were not aware that it was these sausages they would evaluate on the test day. Panel performance was assessed using Tucker1 plot and profile plots in PanelCheck (version 1.4.0, Nofima, Ås, Norway).

Based on the results obtained during the 2 days of training, the final set of attributes and an evaluation procedure were established. A total of 23 attributes in the following categories were selected: consistency in hand, odor from the interior surface, appearance of the exterior surface, appearance of the interior surface, flavor and taste, and finally consistency in the mouth in the first bite. The attributes in each category are listed in Table 2 together with a description of the evaluation procedure for each attribute.

**Table 2:** Sensory attributes arranged into categories with the letter in parentheses representing the attribute category.

Attribute(s)	Category	Evaluation procedure
Break	Consistency in hand (H)	Hold the sausage in each end, snap it against its curvature in an upward direction and assess how easily the sausage breaks
Sausage Smoky Spicy Cereal	Odor from the interior surface (O)	Immediately after the snap, smell the interior surface of the sausage and assess the intensity of odor
Brown Pricked Unevenness	Appearance of the exterior surface (E)	Look at the exterior surface and assess the appearance
Brown Structure	Appearance of the interior surface (I)	Look at the interior surface and assess the appearance
Salty Spicy Bitter	Taste (T)	Take a bite of the sausage and assess the taste
Smoky Sausage Cereal	Flavor (F)	Take a bite of the sausage and assess the flavor
Crispness Juiciness Fatty Firmness Crumbliness Grainy Chewing time	Consistency in the mouth (M)	Take a bite with the front teeth and assess the crispness Chew the sausage five to six times and assess the moisture release While chewing, assess the texture attributes Chew the sausage and assess the duration until sample is ready for swallowing

Each attribute was evaluated according to the described evaluation procedure.

Prior to serving, the sausages were cooked in a hot steam oven (Air-O-Steam, model AOS10EA; ELX, Pordenone, Italy) at 100% humidity for 4 min until a core temperature of 68–70°C was reached. The temperature was controlled using a cooking thermometer (Testo 926; Buch & Holm, Herlev, Denmark) inserted lengthwise into one of the sausages for each heating. The sausages were sliced into 10 cm thick pieces, and these were placed on a preheated porcelain plate marked with a three-digit sample number and then covered by a foil tray and served hot.

To reduce session-to-session variation, all sausages were evaluated on the same day, and, because only five samples were evaluated, three replicates per sausage were considered acceptable. The sausage order was randomized in three blocks using FIZZ software (Biosystemes, FIZZ, Sensory Software version 2.46B, Copyright 1994–2010, Couternon, France), and the sausages were served in the same order to all panelists. To reduce the effect of sensory fatigue, there was a short break halfway through the profiling.

The panelists ate maximum two bites of each sample. Between samples the panelists were asked to cleanse the palate by drinking water and eat a piece of melon or flat bread if needed. The sensory attributes were evaluated objectively using an unstructured 150 mm line scale with an anchor of 1 cm from each side, with the endpoints “low” and “high” intensity attached to each end.

### Consumer tests

The two sausages with added bran, RBS, and WBS, were evaluated in two consumer tests: a central location test (CLT) in an after-school club for 6- to 9-year-old children and a home use test (HUT) for the children's parents. In the two consumer tests, a total of 40–60 consumers per segment were considered acceptable (Resurreccion 1998). These two consumer groups were selected because they represent a relevant target group for healthier sausages since it is a convenient product that is eaten by young families in Denmark. The number of samples included in the consumer test was limited to three samples and therefore the commercial sausage with 10% w/w fat (10%S) was included as the control. Also, this sausage represented the competing product on the market. In the consumer test, liking and rating of holistic words were measured. The cooking procedure was same for both consumer tests: the sausages were added to a pot of boiling water, which was then removed from the heat source, and the sausages were heated for 10 min. Colored labels were used to identify the three test sausages to the participants and the respective questionnaires were printed on paper in the same color. Hereby it was ensured that all of the participants were blinded to the type of sausage being evaluated while filling out the correct questionnaire.

In the CLT with the children, a 5-point smiley scale was used to indicate liking. Furthermore, the children were asked to point out which set of depicted foods could be associated with each sausage. The depicted foods were wheat bread, carrot, steak, sausages, broccoli, cereal, tomato, hotdog, candy, rye bread, and potato. A blank space was provided for the children to draw additional associations that were not depicted. This was a nonverbal way of assessing the associations experienced by the children when tasting the sausages. To ensure valid responses, the children were divided into small groups, and, before tasting the sausages, they were thoroughly informed about the test and instructed on the use of the scale and the drawings. The children were presented to one sample at a time consisting of one half of the sausage (about 10 cm) in a random order. The children were encouraged to evaluate the samples individually while not talking to each other.

In the HUT, the parents were given a bag containing one piece of each of the test sausages for home evaluation and a document describing the cooking and evaluation procedure. The sausages were evaluated in a 7-point categorical scale with boundary descriptors ranging from “do not like at all” to “like very much” used to indicate liking. Furthermore, the parents were asked to rate a list of holistic words on a 150 mm unstructured line scale with boundary descriptors ranging from “little” to “much” according to “Holistic by DMRI,” which is a rapid method developed by DMRI and used to assess consumers' sensory perception of a given product. The holistic words in this test were based on previous studies with meat products and included the following words: appealing, harmonious, complex, delicious, healthy, natural, odd, confusing, and cozy. Test results from “Holistic by DMRI” were converted into numerical scores by measuring the distance from the left end of the scale to the point marked by the consumer.

### Statistical analysis

Sensory profiling data were collected using the FIZZ software (Biosystemes, FIZZ, Sensory software version 2.46B, Copyright 1994–2010), and unstandardized data were analyzed with the full cross-validated multivariate method principal component analysis (PCA) using Unscrambler (version 9.8, CAMO Software, Oslo, Norway).

Furthermore, using PanelCheck (version 1.4.0, Nofima) a two-way analysis of variance (two-way ANOVA), with sausage as fixed effect and the interaction assessor × sausage as well as assessor as random effect was performed.

The Bonferroni least significant difference (LSD) and a significance level of *P* ≤ 0.05 were used to indicate statistically significant differences. All panelists received the sausages in the same order, but any potential carry-over effect or sensory adaptation was taken into account by randomizing the five sausages for each replicate. In a three-way ANOVA including a test for the replicate effect, there was only significant difference between samples on one attribute (brown interior color). Therefore, analysis of data was conducted using a two-way ANOVA. Panelist performance was assessed using profile plots from PanelCheck (version 1.4.0, published 2010).

In the consumer test, the liking data were visualized using Microsoft Office Excel (version 14.0.6112.5000, published 2010). A PCA with full cross-validation was performed on the unstandardized data of the parents' ratings of hedonic words using Unscrambler (version 9.8). One-way ANOVA was performed to assess differences between sausage samples using the Bonferroni LSD and *P* ≤ 0.05 indicated statistical significance.

The multivariate analysis partial least squares regression (PLSR) in Unscrambler (version 9.8, CAMO Software) was used to plot objective sensory data from the sensory profiling as independent variables (*Y*) and the nutritional data standardized according to the standard deviation as predictor variables (*X*).

## Results

### Sensory profiling

**Figure 1:**
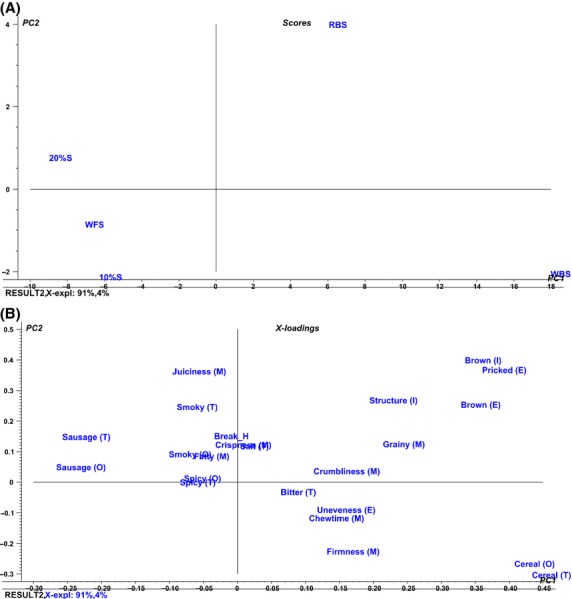
Principal component analysis (PCA) of the data from the sensory profiling using a trained sensory panel (*n* = 9). (A) PCA scores presenting the five sausages: wheat bran sausage (WBS), rye bran sausage (RBS), wheat flour sausage (WFS), sausage with 20% w/w fat content (20%S), and sausage with 10% w/w fat content (10%S). (B) PCA loadings of the evaluated sensory attributes. Letters in parentheses indicate the attribute category: consistency in hand (H), odor from the interior surface (O), appearance of the exterior surface (E), appearance of the interior surface (I), taste (T), flavor (F), and consistency in the mouth (M).

The results from the PCA of the sensory profiling data are presented in score and loading plots in Figure 1. The score plot shows the sausages with added bran (RBS and WBS), WFS, and also the two commercial sausages 20%S and 10%S (Fig. 1A). As seen in Table 3, 21 of 23 attributes were significant in describing the differences between the sausages (*P* ≤ 0.05). Overall principal components (PC) 1 and 2 covered 95.5% of the total variability in data, with the horizontal PC1 being the component with the highest explanatory power (91.0%). PC1 explained the difference between the sausages with added bran (RBS and WBS) and the sausages without bran (WFS, 10%S, and 20%S). The two sausages that differed most were 20%S and WBS, and the difference could be explained by taste and odor attributes: 20%S was highly correlated with sausage taste, and odor, while WBS was correlated with cereal taste and odor (Fig. 1 and Table 3).

**Table 3:** Mean intensity ratings of sensory attributes of sausages (using an unstructured line scale from 0 to 15 mm).

	RBS	WBS	WFS	20%S	10%S	Bonferroni LSD	*P*-value
Break (H)	7.0	6.0	7.4	7.9	6.1	2.4	0.112
Sausage (O)	5.1^b^	1.9^c^	8.9^a^	9.7^a^	7.9^a^	1.9	<0.001
Smoky (O)	6.2^a^	4.0^b^	7.2^a^	7.0^a^	7.0^a^	1.5	<0.001
Spicy (O)	5.2^a^	3.7^b^	6.2^a^	5.8^a^	6.3^a^	1.5	<0.001
Cereal (O)	4.7^b^	11.6^a^	0.0^c^	0.0^c^	1.2^c^	2.4	<0.001
Brown (E)	9.1^b^	11.2^a^	3.6^c^	3.3^c^	3.6^c^	2.0	<0.001
Pricked (E)	9.3^a^	11.0^a^	2.8^b^	2.4^b^	2.8^b^	2.2	<0.001
Uneven (E)	3.1^b^	5.5^a^	2.1^b^	3.0^b^	2.2^b^	1.9	<0.001
Brown (I)	8.6^a^	9.8^a^	2.3^b^	2.0^b^	2.2^b^	2.5	<0.001
Structure (I)	7.7^a^	8.2^a^	4.0^b^	4.1^b^	3.7^b^	2.6	<0.001
Salt (T)	8.6	7.4	7.5	7.7	8.2	1.7	0.166
Spicy (T)	7.7^b^	5.6^c^	6.9^b,c^	8.0^b^	10.0^a^	1.5	<0.001
Bitter (T)	3.1^a,b^	3.6^a^	1.9^b^	2.0^a,b^	3.0^a,b^	1.7	0.021
Cereal (F)	4.7^b^	12.3^a^	0.0^c^	0.0^c^	1.1^c^	2.4	<0.001
Sausage (F)	6.1^b^	2.3^c^	9.3^a^	10.0^a^	8.1^a^	2.0	<0.001
Smoky (F)	8.2^a^	4.9^b^	7.9^a^	7.8^a^	8.1^a^	1.8	<0.001
Crispness (M)	7.2^a,b^	6.5^b^	7.5^a,b^	8.5^a^	6.6^b^	1.9	0.02
Juiciness (M)	7.5^b^	4.8^d^	7.6^b^	9.2^a^	6.1^c^	1.3	<0.001
Fatty (M)	2.1^b^	1.3^b^	2.1^b^	4.5^a^	2.5^b^	1.4	<0.001
Firmness (M)	6.9^b^	8.9^a^	6.8^b^	3.9^c^	6.7^b^	1.5	<0.001
Crumbliness (M)	5.9^a,b^	6.5^a^	4.4^a,b^	3.5^b^	4.3^a,b^	2.6	0.008
Grainy (M)	4.6^a^	6.2^a^	1.3^b^	1.0^b^	1.3^b^	2.4	<0.001
Chewing time (M)	6.2^a,b^	7.3^a^	5.3^b^	3.8^c^	5.8^b^	1.3	<0.001

Letters in parentheses indicate the attribute category. Bonferroni LSD is used as a measure for significant difference between samples when performing multiple comparisons, thereby decreasing the risk of type I error (false positive repsonse). *P* ≤ 0.05 indicates significant differences and different letters in each row indicate significant differences. RBS, rye bran sausage; WBS, wheat bran sausage; WFS, wheat flour sausage; 10%S, 10% fat sausage; 20%S, 20% fat sausage; H, consistency in hand; O, odor from the interior surface; E, appearance of the exterior surface; I, appearance of the interior surface; T, taste; F, flavor; M, consistency in the mouth.

### Texture

An interesting pattern was revealed when analyzing the sensory data with respect to texture. As can be seen in Table 3, no significant differences between the sausages with regard to consistency evaluated by breaking the sausage against its curvature were found (*P* = 0.112). However, crispness and juiciness were particularly high for 20%S, as opposed to WBS and 10%S, which were given the lowest ratings in these two parameters. Interestingly, RBS and WFS were not significantly different from 20%S with respect to crispness. Furthermore, RBS and WFS were equally juicy although slightly less juicy than 20%S, but higher than 10%S and WBS. Generally, fatty mouth-feel was low in all sausages with a fat content of 10% (WBS, RBS, WFS, and 10%S) as opposed to 20%S, which had a high rating in fatty mouth-feel. Firmness and crumbliness were particularly high for WBS followed by WFS, RBS, and 10%S, whereas 20%S received the lowest rating in these two attributes. The sausages without added bran (WFS, 10%S, and 20%S) were correlated with low chewing time before swallowing and a low degree of grainy texture in contrast to WBS and RBS, which had relatively high ratings in these parameters.

### Odor

WFS, 20%S, and 10%S were relatively similar with respect to a high rating in sausage odor, while RBS and WBS received lower ratings for this attribute. Furthermore, WBS differed from the rest of the sausages with respect to smoky and spicy odor, which was low in intensity for WBS, but high in intensity for RBS, WFS, 20%S, and 10%S. In contrast, WBS differed significantly from all the other sausages by having the highest score in cereal odor.

### Appearance

The sausages with added bran and the other sausages could be distinguished from each other in terms of appearance (Table 3, Fig. 1). WFS, 20%S, and 10%S were similar in appearance, specifically in relation to the low intensity of brown color of both the interior and exterior surface and the intensity of pricked appearance and structure. The two sausages with added bran, RBS, and WBS, were similar in appearance and received high ratings for pricked exterior surface and inner structure as well as the intensity of the brown color, although WBS received the highest intensity rating for the exterior brown color. Furthermore, WBS had the most uneven exterior surface, whereas RBS was similar to WFS, 20%S, and 10%S in this attribute.

### Flavor and taste

Similar to the results for sausage odor, sausage flavor was highest for WFS, 20%S, and 10%S, while RBS and WBS received lower ratings for this attribute. Furthermore, cereal flavor was highest for WBS, followed by RBS and finally WFS, 20%S, and 10%S, which received scores close to zero. Salt taste did not differ significantly in any of the sausages (*P* = 0.166), and bitter taste was highest for WBS, although this could not be distinguished from the bitter taste of RBS, 20%S, or 10%S. Furthermore, WBS differed from the rest of the sausages with respect to smoky flavor, which was low in intensity for WBS but high in intensity for RBS, WFS, 20%S, and 10%S. Finally, 10%S differed from all the other sausages by having the most intense spicy flavor (the sensation in the mouth when eating chili or peppercorns), followed by RBS, WFS, and 20%S, and finally WBS.

### Summary of sensory attributes

Of the two sausages with added bran, RBS was the one most similar to the high fat sausage, 20%S, and there was no significant difference between these two sausages with regard to the following attributes such as smoky odor and taste, spicy odor and taste, uneven exterior surface, bitter taste, crispness, and crumbliness. It appeared that the taste and odor of sausage were negatively correlated with cereal taste and odor. The same applied to crispness, which was negatively correlated with firm and grainy. In many cases, the differences between WBS and the sausages without added bran were explained by these attributes, thus representing two contrasting groups.

### Effect of nutritional content on sensory texture attributes

**Figure 2:**
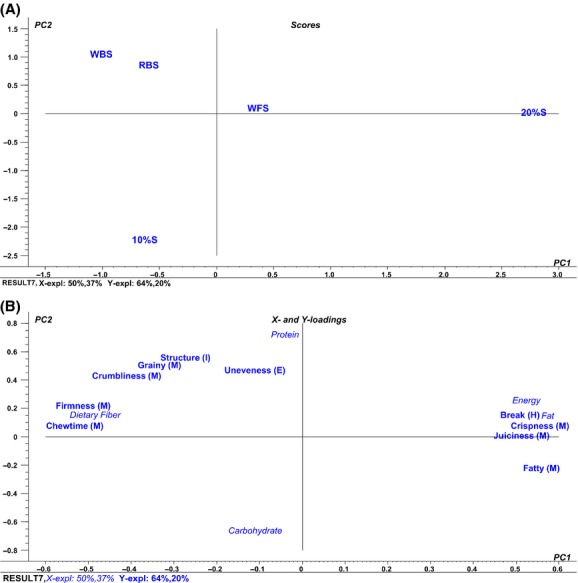
Partial least squares regression (PLSR) of the data from the sensory profiling and nutritional content. (A) PLS scores presenting the five different sausages: wheat bran sausage (WBS), rye bran sausage (RBS), wheat flour sausage (WFS), sausage with 20% w/w fat content (20%S), and sausage with 10% w/w fat content (10%S). (B) PLS *X*- and *Y*-loadings. Nutritional data are included as *X*-loadings and are standardized. Letters in parentheses indicate the attribute category: consistency in hand (H), odor from the interior surface (O), appearance of the exterior surface (E), appearance of the interior surface (I), taste (T), flavor (F), and consistency in the mouth (M).

Based on the sensory profiling, it can be hypothesized that the nutritional content of the sausages has a considerable impact on the sensory attributes and, furthermore, that fiber versus fat content is a very important determinant. Therefore, a PLSR was performed to study the extent to which the variation in nutritional content could explain the result of the sensory attributes, by plotting nutritional data as *X*-loadings and sensory data as *Y*-loadings. Altogether, the nutritional data explained 87% of the variation, and the sensory attributes explained 84%. Most of the variation in both loadings could be explained horizontally. When including only the attributes directly or indirectly related to texture, the dietary fiber content was inversely associated with energy and fat content in the horizontal direction (Fig. 2). Furthermore, these variables could predict differences in some of the textural properties. Dietary fiber was correlated with firmness and chewiness, whereas energy and fat were correlated with how easy the sausage would break and furthermore the crispness, juiciness, and fatty mouth-feel. The former attributes were descriptive of RBS and WBS, whereas the latter were descriptive of the commercial 20%S.

### Consumer tests

**Figure 3:**
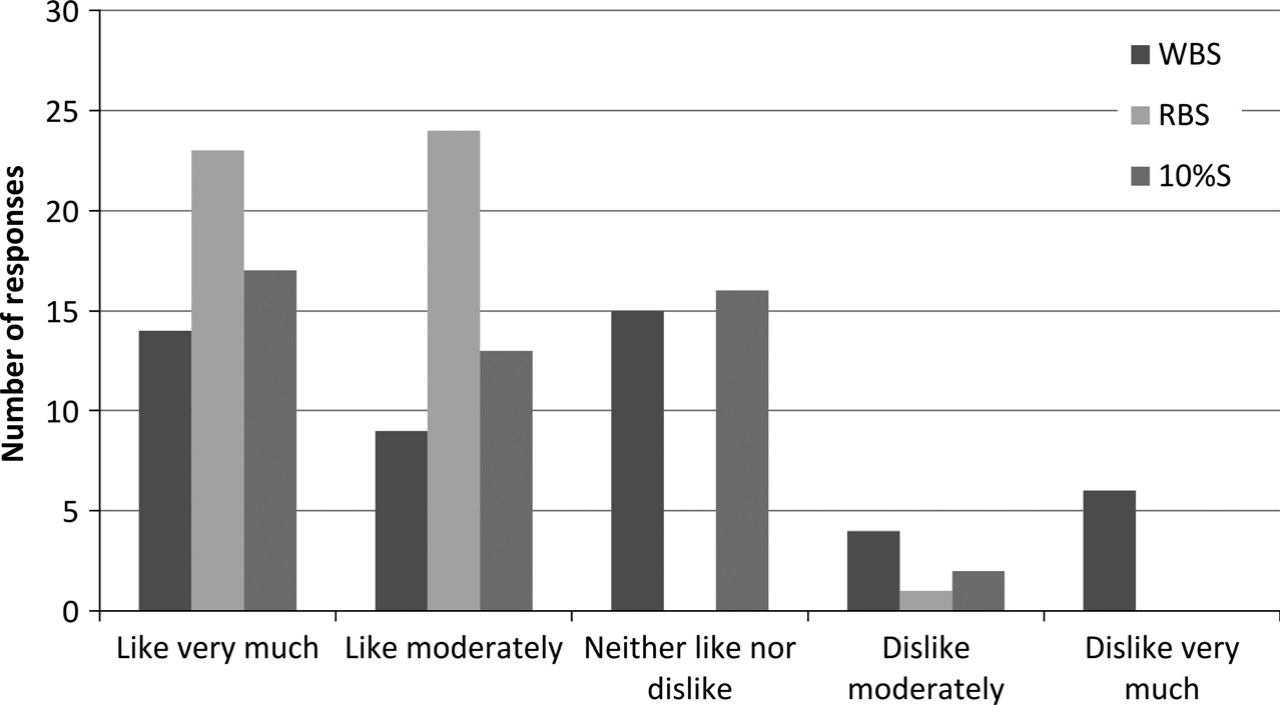
Liking of wheat bran sausage (WBS), rye bran sausage (RBS), and sausage with 10% w/w fat content (10%S) evaluated by children in central location test (*n* = 49). Liking was evaluated on a 5-point smiley scale.

A total of 49 children (20 boys and 29 girls, aged 8.5 ± 1 year) participated in the CLT. The degree of liking rated by the children is shown in Figure 3. RBS was mainly given two positive smileys, indicating a high degree of liking. Liking scores for 10%S were evenly distributed among the two positive smileys and the neutral smiley, which was also the pattern for WBS. WBS was the only sausage for which the most negative smiley was used. For the statistical analysis, the smileys were converted to values from 1 to 5 with higher values expressing higher liking. The mean values were as follows (mean ± SE): WBS 3.4 ± 0.14, 10%S 3.9 ± 0.14, and RBS 4.4 ± 0.14. The statistical test showed that liking of RBS was significantly higher than liking for both WBS and 10%S (*P* < 0.001 and *P* = 0.017, respectively) and furthermore liking for 10%S was significantly higher than for WBS (*P* = 0.017).

In the part of the test where the children evaluated pictures that could be associated with the sausages, WBS was the only sausage associated with cereal (*n* = 7) and bread (*n* = 3), and a relatively large proportion of the children (*n* = 29) even added a drawing of pasta to the blank space. This was in contrast to the RBS and the commercial sausage, 10%S, which was associated with pictures of sausages (*n* = 34 and 20, respectively), steak (*n* = 13 and 10, respectively), and hotdog (*n* = 13 and 9, respectively). A few of the children (*n* = 5) drew chili or pepper when evaluating 10%S.

**Figure 4:**
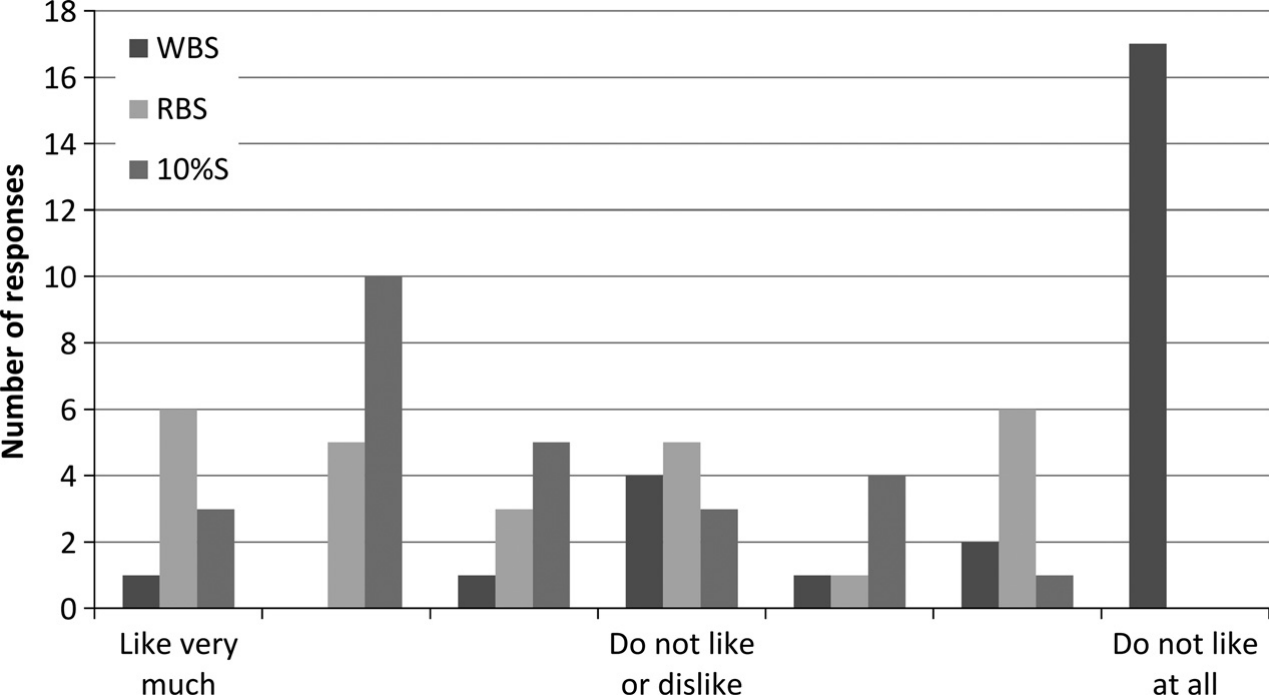
Liking of wheat bran sausage (WBS), rye bran sausage (RBS), and sausage with 10% w/w fat content (10%S) evaluated by parents in home use test (*n* = 24). Liking was evaluated on a 7-point categorical scale.

**Figure 5:**
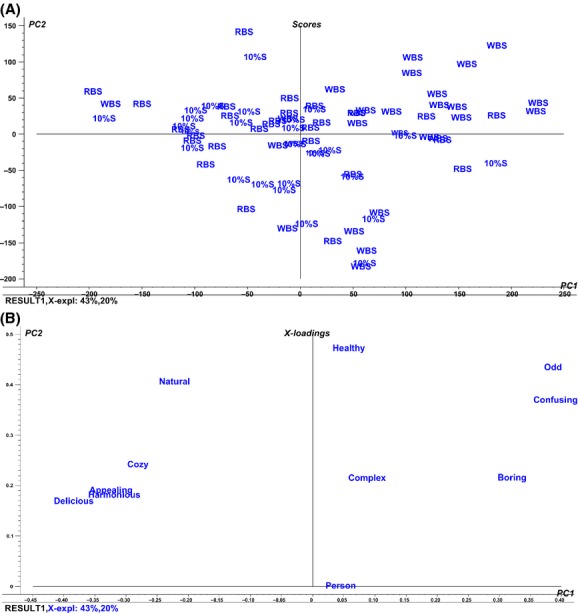
Principal components analysis (PCA) of holistic data from the consumer evaluation including parents (*n* = 24). (A) PCA scores presenting the three sausages: wheat bran sausage (WBS), rye bran sausage (RBS), and sausage with 10% w/w fat content (10%S). Each observation represents an individual consumer. (B) PCA loadings of the 11 holistic words.

A total of 30 questionnaires were collected from the parents, three of whom had not answered the background questions, and a further three were excluded because their answers indicated that they were not parents. Altogether, 24 parents (11 women and 13 men, aged 43 ± 7 years) were included in the HUT. The parents came from different households, and the demographic data are presented in Table 4. These data show that they were a relatively well-defined segment, with the majority having more than one child. Most of the children were aged between six and nine, and these were the ones studied in the CLT. Furthermore, they were highly educated parents. The serving of sausages (e.g., barbecue sausages, wiener sausages, frankfurters, or chicken sausages) took place one to two times per month, although many parents reported that this can vary depending on the season due to the popularity of barbecue sausages in the summertime. The consumer test for the parents showed a similar pattern for ratings of liking to that of the consumer test for the children (Fig. 4). WBS was by far the least liked sausage, and it was the only sausage given the most negative rating by the majority of the parents. Liking scores for 10%S and RBS were evenly distributed in the positive end of the scale, although it seemed that 10%S was given a slightly higher proportion of positive ratings. For the statistical analysis, the smileys were converted to values from 1 to 7 with higher values expressing higher liking. The mean values were as follows (mean ± SE): WBS 2.2 ± 0.28, 10%S 5.2 ± 0.28, and RBS 4.9 ± 0.28. Although only 24 consumers participated in the test, significant differences were seen. Liking of WBS was significantly lower than for RBS and 10%S (*P* < 0.001 for both sausages), whereas liking of RBS and 10%S did not differ significantly (*P* = 0.53). Figure 5 shows PCA scores and loadings from ratings of holistic words. Since only three sausages were included in this part of the analysis, each consumer was plotted as a separate observation. PC1, which represents 43% of the variation in the dataset, explained the biggest variation, distinguishing RBS and 10%S from WBS. WBS was correlated with the words “confusing” and “odd,” in contrast to “harmonious,” “delicious,” “cozy,” and “appealing,” which correlated with 10%S and RBS. RBS was correlated with “natural” to a greater extent than WBS. RBS and WBS differed from 10%S in that they both were correlated with “healthy.” PC2 explained 20% of the variation in the dataset, which might reflect differences between RBS and 10%S. The words “appealing,” “harmonious,” and “delicious” might be more closely correlated with 10%S, although this seems to be highly dependent on the particular consumers' rating.

**Table 4:** Demographic information on parents in the HUT and frequency of serving sausages in the home.

Gender (n)	
Male	13
Female	11
Age	
Mean ± SD	43 ± 7 years
Education of parent	
Elementary school or equivalent (7–10 years' school attendance)	4.2% (*n* = 1)
Apprenticeship or trained in a profession	0.0%
High school education (∼12 years' school attendance)	0.0%
Short-cycle higher education (1½–2½ years)	8.3% (*n* = 2)
Undergraduate study (3–4 years)	54.2% (*n* = 12)
Graduate study (3–8 years)	33.3% (*n* = 8)
Number of children in the household	
One child	4.2% (*n* = 1)
Two children	54.2% (*n* = 12)
Three children	37.5% (*n* = 9)
Age of the children	
0–5 years	33.3% (*n* = 8)
6–9 years	87.5% (*n* = 20)
11–15 years	29.2% (*n* = 7)
16 years or above	12.5% (*n* = 3)
Frequency of serving of sausages in the household	
Every day	0.0%
One to two times a week	0.0%
One to two times a month	70.8% (*n* = 16)
A couple of times a year	25.0% (*n* = 6)
Rarely	4.2% (*n* = 1)
Never	0.0%

## Discussion

The present study represents a unique combination of sensory profiling data and consumer liking of sausages that were nutritionally improved by the addition of rye or wheat bran. The aim of the study was to investigate whether the addition of dietary fiber via wheat or rye bran to sausages with 10% w/w fat affected sensory properties and consumer liking. The results showed that the addition of bran to the sausages affected the sensory characteristics of the sausages. RBS and WBS differed significantly from the other test sausages in that they were browner, more pricked, and had a coarser inner structure. Of the two sausages with added bran, RBS was the one most similar to the high-fat commercial sausage (20%S). The assessment of consumer liking showed that liking was lower for WBS than for RBS, and the latter was not assessed as different from the commercial 10%S. RBS was correlated with more positive holistic words than WBS***.***

### Firmness and chewing time

As seen from our results, the fiber source could be an important determinant for firmness, with wheat bran increasing firmness as opposed to rye bran, compared with a sausage with no bran but the same fat content. Since no other studies have made a direct comparison between wheat and rye bran as sources of dietary fiber, discussion of the fiber source is limited to the results from the present study.

It could be suggested that the increased firmness of WBS could be attributed to the content of nonsoluble fiber from wheat bran, which also results in a prolonged chewing time. This is consistent with the results of Yılmaz (2005), who found that the addition of 20% wheat bran increased the firmness of meatballs, although no significant differences were seen in sausages with wheat bran content below this level. Szczepaniak et al. (2005) investigated the addition of 7.5% and 10% pure wheat fiber to sausages but found no effect on hardness.

In this study, the firmness of RBS was not different from the firmness of WFS, which is a comparable sausage with respect to dietary composition, although it does not contain any bran. There is not enough evidence or studies on the effect of overall soluble fiber (e.g., rye or oat) or rye bran on the firmness of meat products to draw any clear conclusions. Generally, it seems that soluble fiber does not increase, but potentially reduces, firmness. This has been shown for the addition of different levels of rye bran to meat balls (Yılmaz 2004), 7.5% or 10% purified oat fiber to sausages (Szczepaniak et al. 2005), 0.3% or 0.8% soluble β-glycan from barley to sausages (Morin et al. 2002), and 1% oat fiber to sausages (Cofrades et al. 2000).

The exact amount of dietary fiber and fat in the sausages appears to be relevant for the results. The effect of rye and wheat bran doses was not investigated in this study but was established in preliminary recipe development, based on achieving a good sensory quality of sausages and a substantial contribution of fiber (data not shown). WBS contained 6.2% wheat bran and ∼3.2% dietary fiber, which is relatively low compared to the studies by Yılmaz and Szczepaniak. Szczepaniak et al. (2005) did not find a difference in the hardness of sausages with up to 10% purified wheat fiber compared with sausages with no added fiber. It is important, however, to take into account the fact that the fat content in these sausages was almost twice as high (18–20% fat) as in the sausages in the present study (10% fat).

Altogether, the results indicated that the addition of a nonsoluble fiber source, such as wheat bran, increased the firmness of sausages, whereas the addition of a soluble fiber source, such as rye bran, did not affect firmness to the same degree compared with sausages with a similar fat content. It was not possible to include fiber type in the PLSR and make a direct analysis of this relationship, due to a lack of data on the precise content of soluble versus nonsoluble dietary fiber in the two bran fractions.

In the present study, WBS and RBS were similar with respect to chewing time before swallowing, whereas WFS had a shorter chewing time. This indicates that the addition of bran, and thereby dietary fiber, contributes to an increased chewing time. Firmness seemed to correlate with chewing time before swallowing, and, furthermore, the PLSR clearly showed that high dietary fiber, low- fat, and energy contents were predictors of these two attributes. No other studies have investigated the effect of rye or wheat bran addition to sausages on chewing time, although this attribute could prove to be important for the development of satiety during a meal. The texture of a food might be important for the development of satiety because the presence of food in the mouth affects the satiety cascade (Blundell 1999). Prolonging oro-sensory exposure to foods might lead to a higher satiety response and earlier meal termination and furthermore, it has been suggested that the concept of texture-specific satiety might be an important component of satiety (Guinard and Brun 1998; de Graaf 2012).

### Juiciness

Many factors could have an impact on the juiciness of the sausages. The contents of fat and soluble dietary fiber from the bran are important factors for consistency. The point at which the amount of added fibers adversely affects the sensory characteristics has not yet been established Elleuch et al. (2011). It is therefore important to perform sensory studies, such as the present study, to investigate consistency, texture, and also consumer liking when adding dietary fiber to meat products. Juiciness is considered an important quality parameter when evaluating consumer liking of meat products such as sausages (Resurreccion 2004). Dietary fibers are only capable of compensating for the reduction in fat content to a certain degree, as was observed in the present study, in which the sausage with added rye bran was rated as being as juicy as a commercial low-fat sausage (10%S) but not as juicy as the commercial high-fat sausage (20%S). These results are in accordance with results obtained by Yılmaz (2004, 2005), who found that substitution of fat with wheat bran above a level of 10% significantly decreased juiciness, while the addition of rye bran in amounts of 5% or 10% did not affect juiciness. Similarly, the addition of the soluble fiber β-glycan in purified form was not found to affect juiciness when added to sausages in amounts of 0.3% and 0.8% (Morin et al. 2002).

### Odor and flavor

Sausage odor and flavor were negatively correlated with cereal odor and flavor, which is consistent with the results obtained by Morin et al. (2002). It is unclear from this study whether the significant differences were only related to the aroma experience or if the appearance could have had an effect on the perceived odor and flavor. This is speculated because the sausages with added bran were also darker than the other test sausages. Future studies could take this issue into account in the study design by masking the sausage color with lighting. Furthermore, the commercial sausages were not manufactured in the same way as the test sausages and the recipes might vary. Therefore, it might be relevant to manufacture the high-fat sausage using the same recipe as for the sausages with bran. In this study, however, it was prioritized to compare with the commercial alternatives with the same fat content; in this case it is 10%S.

The fiber contents of RBS and WBS varied to a certain degree, possibly due to cereal variation, differences in sausage drying loss, or analytical error. Nevertheless, the differences in sensory results between RBS and WBS are believed to be due to the different proportions of soluble and nonsoluble dietary fiber in rye and wheat bran.

### Consumer acceptance

Of the two sausages with bran, RBS was rated as the most liked, while WBS was rated as the least liked. Furthermore, the liking of RBS was similar to the liking of the commercial 10%S, and RBS might therefore represent a competitive alternative to this commercial sausage. Other studies have found that the addition of rye bran in amounts of up to 15% or wheat bran in amounts of up to 5% does not affect the palatability of low-fat meatballs (Yılmaz 2004, 2005). Interestingly, the WBS was clearly distinguished from the RBS and 10%S, and this was reflected in the pictures the children associated with it (cereal, bread, and pasta). Furthermore, it was consistent with the sensory profiling, in which WBS also had the highest rating in cereal odor and flavor. Of particular note is the similarity between RBS and 10%S and the fact that RBS seemed to perform well among the consumers, especially children.

The CLT with the children was conducted under controlled test conditions, whereas the HUT with the parents was conducted under less stringent test conditions, thereby resulting in a more natural environment for tasting and evaluating the sausages and this might have affected the outcome. Generally, the HUT gives higher ratings of liking than the CLT, but the ranking of products does not change (Meiselman 2008). Only relative ratings of liking in the present two consumer tests can be compared, since they were measured by two different target groups using two different scales. The results of liking indicate that the children seemed to prefer RBS to 10%S or WBS, whereas the parents seemed to prefer 10%S. Nevertheless, both consumer groups seemed to agree with respect to a low liking of WBS and the fact that RBS was almost similar in liking to 10%S. Finally, it should be noted that the consumer data obtained in this study only reflect a small proportion of the consumers who regularly consume sausages, and therefore in future studies it would be relevant to investigate consumer acceptance in other consumer groups with different ages, socioeconomic backgrounds, geographical locations, and nationalities.

Interestingly, it seems that the content of viscous dietary fiber in rye bran has both sensory and nutritional benefits, providing a texture similar to that of commercial sausages and also an improved effect on appetite parameters (Vuholm et al. 2014).

## Conclusion

In conclusion, the addition of dietary fiber from rye bran to sausages represents a promising approach toward improving the health benefits of conventional sausages while maintaining consumer liking. In contrast, the addition of wheat bran results in sausages with significantly affected sensory characteristics, resulting in low consumer liking. The results from this study could be used to investigate how the addition of other sources of viscous dietary fiber, such as oats or barley, might affect the sensory attributes of sausages.
